# Application of a Liquid-Liquid Microextraction Method Based on a Natural Hydrophobic Deep Eutectic Solvent for the Extraction of Plastic Migrants from Kombuchas

**DOI:** 10.3390/molecules27010178

**Published:** 2021-12-28

**Authors:** Antonio V. Herrera-Herrera, Ruth Rodríguez-Ramos, Álvaro Santana-Mayor, Bárbara Socas-Rodríguez, Miguel Ángel Rodríguez-Delgado

**Affiliations:** 1 Departamento de Química, Unidad Departamental de Química Analítica, Facultad de Ciencias, Universidad de La Laguna (ULL), Avenida Astrofísico Francisco Sánchez, s/n, 38206 San Cristóbal de La Laguna, Spain; rrodrira@ull.edu.es (R.R.-R.); asantanm@ull.edu.es (Á.S.-M.); bsocasro@ull.edu.es (B.S.-R.); 2 Instituto Universitario de Bio-Orgánica Antonio González, Universidad de La Laguna (ULL), Avda. Astrofísico Fco. Sánchez, 2, 38206 San Cristóbal de La Laguna, Spain

**Keywords:** kombucha, liquid-liquid microextraction, deep eutectic solvent, green solvent, phthalate acid esters, liquid chromatography

## Abstract

A vortex-assisted liquid-liquid microextraction, based on a natural hydrophobic deep eutectic solvent made from the monoterpene thymol and octanoic fatty acid, was employed for the analysis of 11 phthalate esters and one adipate in kombucha (a tea-based fermented beverage). Separation and determination were performed using an ultra-high performance liquid chromatography (UHPLC) system coupled to a single quadrupole mass spectrometer. Confirmatory analyses were carried out through UHPLC tandem mass spectrometry. The full method was validated in terms of matrix effect, matrix-matched calibration, sensitivity, recovery, limits of detection and quantification and repeatability. Satisfactory determination coefficients for quadratic calibration curves (≥0.9938), recovery values (67–120%) and limits of detection (0.07–5.45 µg/L) were obtained. Analysis of 26 kombucha samples reported concentrations for dibutyl phthalate and dimethyl phthalate in the range between the limit of quantification (LOQ) and 16.18 ± 1.14 µg/L, although these phthalates were also detected under the LOQ in some of the analyzed samples. Only one of the samples bottled in plastic containers (7) did not present residues while only five of the 19 samples in glass bottles contained any plasticizer. However, the highest concentration was found in a kombucha bottled in food-grade glass. This work represents the first application in which phthalates and adipates are analyzed in kombuchas.

## 1. Introduction

Green chemistry, also known as sustainable chemistry, aims to design chemical protocols and substances that eliminate (or reduce as much as possible) the generation of hazardous materials [1]. Chemical processes often use significant amounts of non-renewable solvents with high polluting power. Under the umbrella of green chemistry, different alternative solvents have been developed [2] to avoid the contamination caused by the traditional ones and their intrinsic toxicity. Among them, low transition temperature mixtures (LTTMs, solvents formed by combinations of compounds and characterized by a low transition temperature between the solid and liquid states) and room temperature ionic liquids (ILs) have gained increasing interest [3]. In this regard, deep eutectic solvents (DESs) have also been widely studied [4,5] due to their superior characteristics (in comparison to ILs). Their nature consists of at least one hydrogen bond donor and one hydrogen bond acceptor interacting through strong hydrogen bonds (although other forces, such as Van der Waals, halogen, alkyl-alkyl and dipole bonds, and other electrostatic interactions are also involved). The melting point of DESs is considerably lower than that of the individual components and lower than expected from the enthalpies of fusion. Strictly speaking, DESs are characterized by a particular molar ratio of the individual constituents. However, there are different mixtures with melting temperatures lower than expected, which are frequently included in this category (although some authors prefer the term “eutectic mixtures”). DESs are cheap, viscous, non-flammable, have low volatility and their synthesis is easy and scalable. In addition, their toxicity is limited and they have good biodegradability. An aspect that should be cautiously considered is their recyclability. Different methods have been tested, including anti-solvent addition, membrane filtration, solid–liquid extraction, liquid–liquid extraction, supercritical fluid extraction, distillation, crystallization, separation due to density differences, etc. [6] Although additional studies are required, most of these methods usually employ high amounts of additional solvents and energy (with the inherent high cost). This problem can be exacerbated in applications employing high volumes of DESs. In this regard, the so-called natural DESs (NaDESs), entirely prepared from natural components (e.g., organic acids, sugars, amino acids, choline derivatives or fats) can be considered aligned with Green Analytical Chemistry principles. Although the first DESs used had a hydrophilic character, the introduction of hydrophobic DESs (HDESs) [7] was a real revolution because of their compatibility with aqueous samples without the need for extra reagents. In fact, NaHDESs have proven to be an interesting alternative in different analytical applications [7,8,9]. It should be mentioned that, despite their inherent advantages, it has been proved [10,11] that the toxicity of certain DESs is higher than the aqueous solutions of the constituents due to a disruption effect on cell walls.

Global connection and development have led to the spread of ethnic foods around the world. In addition to their particular organoleptic characteristics and the possibility of using them in the fight against famine, their alleged and assumed health benefits are often behind this expansion. In this regard, Kombucha (a probiotic fermented drink) is attributed with therapeutic effects including anti-inflammatory, anticancer, antihypertensive, antidiabetic, hepatoprotective, and antimicrobial properties [12]. Moreover, additional preventive benefits have also been reported, such as delayed aging, improvement of gastrointestinal and glandular functions, blood detoxification and positive cholesterolemic effects [12,13]. These effects, attributed to the presence of different bioactive compounds (e.g., catechins, essential oils, flavonoids, organic acids, phenolic compounds and tannins) and to the symbiotic colony of bacteria and yeasts (SCOBY), remain uncertain as they have not yet been confirmed in humans [12]. Kombucha is thought to have originally appeared in China between 200 and 300 BC. For centuries it was a well-known (and even religiously tinged) beverage in Asian countries and spread around the world via trade routes. In the last two decades, it has become very popular all over the world. In fact, the global market size exceeded $2.6 billion in 2020 and the compound annual growth rate between 2021 and 2027 is estimated at over 17% [14]. Although different definitions have been proposed, kombucha is generally identified as a beverage obtained by fermentation of black, green, or oolong tea (although other substrates, such as sugar beet molasses and sweetened herbal extracts of mint, thyme, rosemary and fennel have also been tested) and sugar using a SCOBY. The result is a slightly sweet and sour sparkling drink (similar to apple cider for short fermentation and with vinegary aromas for long fermentation). Although the production was initially a kind of artisanal activity, today there are different companies producing commercial kombuchas with different varieties of flavor and ingredients. It is worth mentioning that some health risks have also been reported, mainly associated with overconsumption, kombucha contaminated with metals, inorganic substances introduced during production, consumption of highly acidic samples and consumption by people with previous illnesses [12]. However, to the best of our knowledge, organic contaminants in kombucha have not been analyzed, despite the growing popularity of this beverage.

Plasticizers have become a major health concern due to their toxic effects. In this regard, phthalate acidic esters (PAEs) can cause alterations of different tissues (kidney, liver, thyroid, reproductive system) and even cancer [15]. Their use in the agri-food industry (food packaging and contact material during processing) is frequent due to the properties they confer to the final material: flexibility, durability, transparency and longevity [16]. However, these compounds are not chemically bonded to the polymeric matrix but penetrate into these chains increasing flexibility. Consequently, and considering their stability and persistence, they are likely to migrate into the final product. In fact, different biomonitoring surveys have detected PAEs residues in human tissues [15,17]. Therefore, hundreds of analytical protocols have been developed for the analysis of these compounds in foods [18]. In this regard, the use of DESs has promoted new sustainable protocols [8,9,19,20,21]. We have recently developed a NaHDES based on thymol (Th):octanoic acid (OctA) (molar ratio 2:1) for vortex-assisted liquid-liquid microextraction (VA-LLME) of PAEs from tonic water (bottled in plastic, glass and metal containers) and cold infusions [9]. For this purpose, factors affecting extraction efficiency (i.e., type and proportions of NaHDES components, sample pH and NaHDES volume) were optimized using a univariate protocol. The method was demonstrated to be precise and repeatable and yielded recovery in the range 71–124% and lowest calibration levels between 0.025 and 1.25 μg/L. From a Green Analytical Chemistry point of view, the method was evaluated applying the Analytical Eco-Scale [22] reaching 66 points (acceptable greenness). This method seems to be a good alternative for the extraction of this type of compound in different beverages, after the corresponding validation. In this respect, commercial kombuchas are typically bottled in food-grade glass (often with plastic caps), stainless steel vessels, or plastic [12] and the analysis of PAEs on them should be of interest due to the increasing consumption of these probiotic beverages.

For all the above, the aim of this work is the evaluation of the content of 11 PAEs (benzylbutyl phthalate, BBP; di-2-n-butoxyethyl phthalate, DBEP; dibutyl phthalate, DBP; dicyclohexyl phthalate, DCHP; di-(2-ethylhexyl) phthalate, DEHP; diethyl phthalate, DEP; di(2-methoxyethyl)phthalate, DMEP; dimethyl phthalate, DMP; di-n-octyl phthalate, DNOP; di-n-pentyl phthalate, DNPP; dipropyl phthalate, DPP) and one adipate (bis(2-ethylhexyl) adipate, DEHA) residues in commercial kombuchas using a VA-LLME based on the NaHDES Th:OctA (molar ratio 2:1). To the best of our knowledge, this is the first time that PAEs and DEHA are analyzed in kombuchas.

## 2. Results and Discussion

### 2.1. UHPLC-MS and UHPLC-MS/MS Methods

A group of 11 PAEs plus one adipate (DEHA) were selected as key compounds in this study. Different international regulatory standards have been established to restrict the presence of these compounds in foods/drinks. In this regard the European Union, via Commission Regulation No 10/2011, has set a specific migration limit of 0.3 mg/kg for DBP, 1.5 mg/kg for DEHP, 18 mg/kg for DEHA, 30 mg/kg for BBP and a default value of 60 mg/kg for those compounds without a specific limit from plastic materials in contact with food [23]. For its part, the US EPA has included some of the selected PAEs (BBP, DBP, DEHP, DNOP and DNPP) in the Phthalates Action Plan [24] to ensure a correct assessment of this type of compounds.

**Figure 1:**
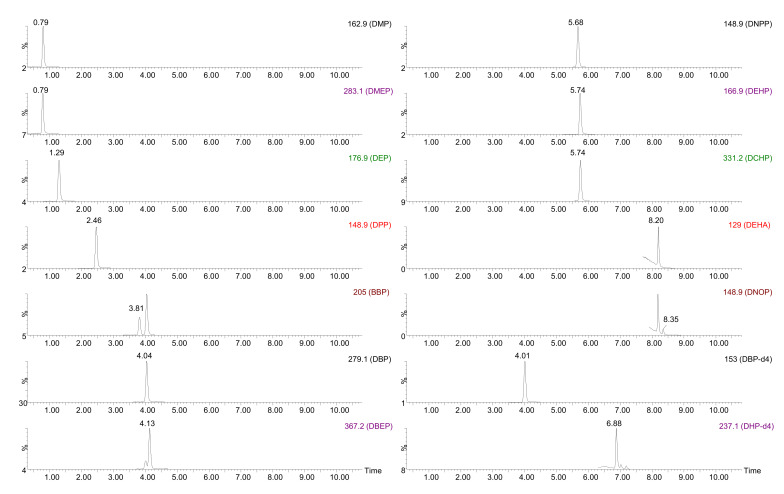
UHPLC-MS chromatogram for a mixture of standards at 150 µg/L. Aqueous mobile phase: 0.1% *v*/*v* formic acid in Milli-Q water. Organic mobile phase: 0.1% *v*/*v* formic acid in acetonitrile. Flow: 0.3 mL/min. MS operated in SIM mode.

In order to guarantee a correct separation and determination of these molecules, and bearing in mind that a single quadrupole (Q) analyzer was used, a UHPLC method previously optimized for a similar group of compounds [25] was selected (fully described in Section 3.2). The mass spectrometer (MS) was initially operated in the full scan mode (using the chromatographic separation) to select the most intense ion for quantification in the single ion monitoring (SIM) mode. Once the optimal conditions were established, the possible contribution to the signal of compounds with close retention times was evaluated and, in that case, new ions were selected. Dibutyl phthalate-3,4,5,6-d_4_ (DBP-d_4_) was used as internal standard (IS) for DMP, DMEP, DEP, DPP, BBP, DBP, DBEP, DEHP, DNPP and DCHP (Rt < 6.0 min) and dihexyl phthalate-3,4,5,6-d_4_ (DHP-d_4_) was employed for DEHA and DNOP (Rt > 6.0 min) due to their similar behavior to those substances. Adequate separation was observed for the target analytes with a total analysis time of 11 min. Figure 1 shows the chromatograms obtained under these conditions for the selected analytes injected at 150 µg/L.

Since the objective of this work is to check the presence of PAEs residues in kombuchas, those samples presenting one of these compounds were also analyzed with a UHPLC-MS/MS instrument using the conditions described by Santana-Mayor et al. [9]. Those conditions are included in Section 3.2. It should be noted that this confirmatory protocol allows ratification of the presence/absence of PAEs in less than 5.0 min. 

### 2.2. VA-LLME of Kombuchas Using NaHDES

The NaHDES Th:OctA 2:1 was previously employed in our laboratory [9] for the extraction of a similar group of PAEs from tonic water and cold infusion with good results. Therefore, it is a promising protocol for the analysis of other beverages. However, considering the complexity of kombuchas with regards to the previously analyzed drinks (with several different ingredients and SCOBY), the method must be fully validated. The samples were firstly centrifuged and filtered to separate the SCOBY. The omnipresent occurrence of PAEs in laboratory material, high-purity solvents and laboratory air dust difficult their analysis [26]. A frequently applied strategy [26] consists of extracting procedural blanks (matrix and Milli-Q water) in each analyzed batch and subtracting peak areas (when necessary) to avoid biased results. 

The properties of NaHDESs composed of terpenes and monocarboxylic acids have been previously studied [27,28]. Martins et al. [27] found that DESs prepared from Th and monocarboxylic acids (including OctA) showed a higher hydrogen-bond acidity character than other typical organic solvents (alcohols ketones, alkanes and aromatics) but slightly lower than that of water. This behavior was almost independent of the alkyl chain length of the acid. Moreover, these NaHDESs presented a higher ability to establish non-specific interactions with solutes than other organic solvents due to the aromatic ring on Th, which also decreases its ability to act as a hydrogen bond acceptor. Thus, although other interactions cannot be ruled out, we hypothesize that the extraction is performed by the formation of π-π interactions between PAEs and NaHDES. This is also in agreement with the fact that, in our previous application [9], better extraction efficiency was observed by using the Th:OctA molar ratio 2:1, since proportionately larger quantities of Th will increase the number of π-π interactions.

#### Validation of the Method

The VA-LLME-UHPLC-MS (Q) method was validated in terms of matrix effect (ME), calibration, sensitivity, extraction efficiency, limits of detection and quantification, and repeatability.

The presence in the matrix of other compounds than analytes could result in an increase or decrease of the signal when chromatography coupled to MS is employed [29]. This fact could be particularly important in these complex samples due to the introduction of the NaHDES components in the final extract. Therefore, a ME study was performed using the method proposed by Matuszewski et al. [30]. For this purpose, the signal for the analytes injected in the matrix (obtained after the application of the extraction protocol) is compared with those signals for the analytes dissolved in a solvent at the same concentration. Signal enhancement is produced when this ratio is greater than 100% while values below 100% indicate signal suppression. This study was performed at 37.5 µg/L. As shown in Table 1, an important ME was observed for all selected compounds (except IS DBP-d_4_, ME: 107%). Only the adipate (DEHA) presented a signal enhancement whereas the rest of the compounds were affected by significant signal suppression (ME: 13–66%). In view of these results, ME compensation is essential for correct quantification.

Matrix-matched calibration is often applied for ME compensation when a blank matrix is available [29]. Therefore, analytes dissolved in the matrix extract were injected in triplicate at 8 increasing concentrations and calibration curves (peak area/IS peak area vs. concentration) were obtained. The use of isotope-labeled ISs as surrogates improves the accuracy by compensating for variations during sample preparation. To check the best fit for the calibration curve (linear or quadratic) the Mandel’s test was applied [31]. This test compares the residual standard deviations of linear and non-linear models by means of an *F*-test. For the calculation of *F* experimental the degrees of freedom for the linear and non-linear models are also used [31]:

(1) $$F_{exp}=\frac{\left(n-2\right)S_{y/x,\;lin}^{2}-\left(n-3\right)S_{y/x,\;cuad}^{2}}{S_{y/x,cuad}^{2}}$$

where *S_y_*_/*x,lin*_ and *S_y_*_/*x,cuad*_ are the residual standard deviations for the linear and quadratic model (respectively) and *n* is the number of levels of concentration. As can be seen in Table 1, *F_exp_* (26.56–6885.32) >> *F_tab_* (6.61 for α = 0.05, numerator degrees of freedom = 1, *n* − 3 = 5). Thus, quadratic curve calibrations with adequate determination coefficients (R^2^), higher than 0.9938, were obtained for the PAEs. The results of this matrix-matched calibration, including equation, ranges tested (sample concentration) and R^2^ are also compiled in Table 1. According to the ISO 8466-2:2001 [32], the instrument sensitivity (e) for quadratic calibration curves can be defined as its first derivative (Table 1), and the sensitivity in the center of the working range (E) is the slope of the tangent to the calibration function in that point (Table 1).

To assess the extraction efficiency of the VA-LLME, a recovery study was carried out at two levels of concentration. Five aliquots of spiked kombucha were extracted and the peak areas were compared with those obtained for the PAEs spiked at the same level in a blank extract. Table 2 shows the results for this study including the concentration levels. Recovery values for the low level (2.5 µg/L, except for DMEP, BBP, DBP and DNOP: 18.75 µg/L) were in the range 67–120% with RSDs between 2% and 19%, while for the high concentration (50 µg/L) recovery ranged between 107 and 120% (RSDs: 3–16%) 

The limits of detection (LODs) and quantification (LOQs) of the method were estimated as those concentrations providing a signal-to-noise ratio (S/N) equal to three or ten, respectively (Table 2). LODs varied in the range 0.07–5.45 µg/L while the LOQs varied from 0.24 µg/L for DNPP to 18.15 µg/L for DMEP. 

The Commission Decision 2002/657/EC expresses repeatability as the coefficient of variation (%) of fortified samples analyzed with the same method on identical test items in the same laboratory by the same operator using the same equipment. The repeatability study was accomplished through five parallel extractions at 50 µg/L. Satisfactory RSDs were obtained (in the range between 3% and 10%).

Validation results demonstrate the applicability of the method for the extraction of PAEs and one adipate from selected probiotic beverages. 

#### 2.3. Occurrence of Plasticizers in Commercial Kombuchas

Once the methodology was validated, a group of 26 samples was analyzed using the VA-LLME-UHPLC-MS method to determine the residues of PAEs and DEHA present in kombuchas marketed in the Canary Islands (Table 3). Among them, 19 samples were stored in glass bottles with plastic caps and seven in plastic containers. Two of the samples bottled in plastic were zero sugar (Table S1). It should be noted that all the samples had the EU organic product label since all the ingredients come from ecological agriculture.

For those samples in which peaks were detected in the SIM mode at the retention times corresponding to the analytes, a *t*-test was applied in order to check whether the areas were statistically and significantly different from those of the blanks analyzed in the same batch. For peaks with areas that were different, the presence was confirmed by UHPLC-MS/MS. Only analytes with areas that were significantly distinct and confirmed by the presence of the two transitions and the established relative intensities between precursor and product ions in them, were quantified using the matrix-matched calibration. As mentioned above, peak areas for blanks were subtracted when necessary. The results of these analyses are shown in Table 3. The expanded uncertainty was calculated according to ISO standard 8466-2:2001 [32]. Five replicates were analyzed for each sample. 

As can be seen, more than half of the samples did not contain any of the analyzed PAEs or DEHA. However, 11 samples contained at least one of the tested plasticizers. In this regard, only DMP and DBP could be found in the selected kombuchas. As expected, almost all kombuchas bottled in plastic (except K26) presented some of these compounds. However, the concentration was below the LOQ for the vast majority of these samples. Only sample K24 (brand I, original flavor) contained a PAE at a quantifiable concentration (DMP: 7.68 ± 1.56 µg/L). In contrast, only five of 19 kombuchas bottled in glass containers had any residue, but DMP was quantifiable in four of them and the highest concentration detected corresponded to one of these samples: K2, 16.18 ± 1.14 µg/L; K9, 3.22 ± 2.24 µg/L; K10, 3.55 ± 2.17 µg/L; K 15, 4.11 ± 2.34 µg/L. These results demonstrate that it is essential to monitor foods/drinks in which that substance is not expected to be found since PAEs can be introduced during the production step or even by the caps. The manufacturing line (home or industrial scale) may be composed of different plastic materials that can be transferred to the final kombuchas due to production conditions (boiling of tea leaves, fermentation at acidic pH for several days, automatic bottling, etc.). Moreover, another aspect that should be considered is storage. Some of the producers clearly indicated that their products must be stored between 2 and 4 °C, but others did not. Some of the samples were stockpiled in freezers at shops but not all of them. Therefore, other factors that can favor the transference of PAES from bottles or caps, such as temperature, light and time since packaging, should be contemplated. Further studies in this direction would complement the findings and allow a more specific explanation.

In 2007 Bošnir et al. [33] conducted a study about the migration of PAES from plastic materials to mineral waters and soft drinks. They found that sample pH and preservatives had an important influence on migration rate. Regarding pH (since kombuchas have no declared preservatives), acidity media increased the migration of PAEs. Among the studied compounds (DMP, DBP, DEP, BBP and DEHP), DMP (followed by DBP and DEHP) presented the higher migration rates in soft drinks (pH < 3). This effect can explain the higher concentrations of DMP and DBP in the studied samples (pH for kombuchas: 2.80–3.25).

**Figure 2:**
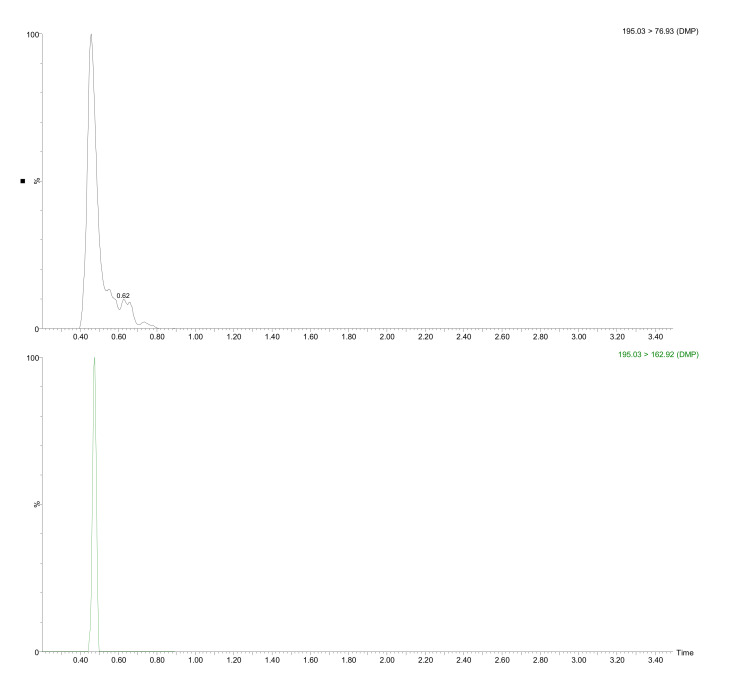
UHPLC-MS/MS chromatogram for detected analyte (DMP) in sample K2 submitted to the developed extraction procedure.

It should be mentioned that no residues of DEHA and DEHP were found (both with restricted limits regulated by the US EPA [34] and WHO [35] in drinking waters. The use of DEHA as an alternative to DEHP is becoming increasingly popular in food contact films and PVC plastics [36]. In addition, its high migration potential [37] and its similar metabolism to DEHP (although more lipophilic), make this compound one of the more hazardous plasticizers. The absence of this compound is good news from a food security perspective. DBP, which migration limit is 0.3 mg/kg in the Commission Regulation No 10/2011 on plastic materials and articles intended to come into contact with food, was detected in five plastic bottles at a concentration lower than LOQ. Figure 2 shows the UHPLC-MS/MS confirmation chromatogram for DMP in sample K2.

Although, as mentioned, there are no previous publications analyzing PAES in kombuchas, since these fermented beverages are prepared from sweetened tea extracts, parallelism can be established with tea drinks (other popular non-alcoholic beverages). In this regard, Wu et al. [38] analyzed 12 tea drinks (sport drinks, coffee drinks and fruit juices were also analyzed) and detected DMP in the range <LOQ–19 µg/L, DEP: <LOQ–76 µg/L, DPP: <LOQ–71 µg/L, DBP: <LOQ; BBP: <LOQ–29 µg/L, and DEHP: 16–123 µg/L. Xue et al. [39] analyzed 105 samples of six different types of drinks and detected DMP: <LOQ–1.30 µg/L, DEP: <LOQ–0.11 µg/L, DBP: <LOQ–3.60 µg/L, BBP: <LOQ–0.19 µg/L, and DEHP: 0.5–12.00 µg/L in tea drinks (22 samples). They also tested the influence of packaging materials (plastic, glass, metal and paper) on the PAEs concentration. Contrary to their expectations (and similar to our results), beverages bottled in paper contained the highest summed concentration of the analyzed PAEs, followed by those bottled in glass, metal and plastic drinks. Apart from a non-representative number of samples for the investigation of the effect of packaging material, the authors explained their results on the basis of the different materials used by different vendors for the same type of plastic package. Also, we detected DEHP in tea drinks in a previous application, but at a concentration below the LOQ [19]. Kombucha samples contained a lower number of PAEs than analyzed tea drinks. Moreover, found concentrations (<LOQ–16.18 ± 1.14 µg/L) were similar [39] or even lower [38] than those found for other tea-based beverages. 

In order to provide an idea of the exposure to DMP (the only quantifiable PAE in these samples) due to the ingestion of kombuchas, the estimated daily intake was calculated through the following equation [40]:

(2) $$EDI=\frac{C\cdot Q}{bw}r_{uptake}$$

where *EDI* (µg/kg-bw/d) is the estimated intake, *C* is the phthalate concentration in the food, *Q* is the average daily ingestion of this food, *bw* is the body weight and *r_uptake_* is the gastrointestinal absorption factor. The European Food Safety Authority [41] and the WHO [42] recommend a daily water intake between 2 and 4 L (80% from beverages and 20% from water in foods [41]). For the calculation, the maximum intake for beverages (80% of 4 L) is considered to come from kombucha. Considering the average concentration of DMP in the samples (6.95 µg/L) and a global average adult weight of 62 kg [43] and assuming a total absorption (*r_uptake_* = 1), the maximum EDI for DMP is 0.36 µg/kg-bw/d. This value is higher than the previously obtained for beverages in China (0.01 µg/kg-bw/d) [40] and Europe (0.00 µg/kg-bw/d) [44]. Although human exposure to DMP from foods is limited (≈10%) [40] its assessment is important since it is an additional source of this low molecular weight PAE to the major one (cosmetics and personal care products) [44].

## 3. Materials and Methods

### 3.1. Chemicals and Materials

Analytical standards and solvents were used as received, without further purification. Acetonitrile (ACN, hypergrade for LC-MS) and hydrochloric acid (25%, *w*/*w*) were bought from Merck (Darmstadt, Germany). Formic acid (for LC-MS) was acquired from Honeywell (Morris Plains, NJ, USA). Sodium hydroxide (≥98%), Th (≥98.5%) and OctA (>98%) were purchased from Sigma-Aldrich Chemie (Madrid, Spain). A Milli-Q A10 gradient system from Millipore (Bedford, MA, USA) was employed to obtain Milli-Q water.

Powdered standards of BBP (CAS 85-68-7), DBEP (CAS 117-83-9), DEHA (CAS 103-23-1), DMP (CAS 131-11-3), DNPP (CAS 131-18-0) and DHP-d_4_ (CAS 1015854-55-3, IS) were acquired from Dr. Ehrenstorfer GmbH (Augsburg, Germany) with a purity of 97% or higher. Standards (purity ≥ 97%) of DBP (CAS 84-74-2), DCHP (CAS 84-61-7), DEHP (CAS 117-81-7), DEP (CAS 84-66-2), DMEP (CAS 117-82-8), DNOP (CAS 117-84-0), DPP (CAS 131-16-8) and dibutyl phthalate-3,4,5,6-d_4_ (DBP-d_4_, CAS 93952-11-5, IS) were provided by Sigma-Aldrich (Madrid, Spain). Appropriate amounts of these compounds were weighed and dissolved in ACN to prepare stock solutions at concentrations in the range between 500 and 1000 mg/L and stored at −18 °C. Working standard mixtures of these solutions were prepared by a combination of stock solutions.

The volumetric material was cleaned with soap and tap water, immersed in Nochromix^®^ oxidizing agent (Godax Laboratories, Cabin John, MD, USA) and rinsed repeatedly with tap and Milli-Q water. The non-volumetric glassware was also cleaned with soap and tap water, rinsed with Milli-Q water and finally pyrolyzed at 550 °C for 4 h. PAEs-free gloves from VWR and pipette tips from Gilson were employed in this work.

### 3.2. Apparatus and Software

Chromatographic separation and determination were performed using an Acquity H-Class ultra-performance liquid chromatography (UPLC^®^) system from Waters (Milford, MA, USA) coupled to an Acquity QDa Q MS (Waters Chromatography, Milford, MA, USA) through electrospray ionization (ESI). This system consisted of a quaternary solvent manager, an automatic sample manager with a flow-through needle and a column oven. The separation was carried out according to the method previously described by Santana-Mayor et al. [25]. Gradient program was applied as follows: 0.0 min 50/50 (*v*/*v*) A/B, 4.0 min 30/70 (*v*/*v*) A/B, 7.0 min 0/100 (*v*/*v*) A/B, 8.0 min 0/100 (*v*/*v*) A/B, 9.0 min 50/50 (*v*/*v*) A/B, 11.0 min 50/50 (*v*/*v*) A/B. Column temperature, mobile phase flow and sample temperature were adjusted to 40 °C, 0.3 mL/min and 10 °C respectively. The injection volume used was 2.5 µL. The Q was operated in SIM mode with positive ionization. Individual compounds were identified according to their specific ions and retention times (Table 4). Optimal cone voltages are also shown in Table 4. ESI capillary voltage and desolvation temperature were established at 1.5 kV and 600 °C.

For confirmatory purposes, a similar Acquity H-Class UPLC system coupled to a Xevo TDQ triple quadrupole (QqQ) MS through ESI was employed. Separation conditions were as reported in [9]. The mobile phase was pumped at 0.3 mL/min and 40 °C under the following program: 0.0 min 20/80 (*v*/*v*) A/B, 1.0 min 0/100 (*v*/*v*) A/B, 2.0 min 0/100 (*v*/*v*) A/B, 3.0 min 20/80 (*v*/*v*) A/B, 5.0 min 20/80 (*v*/*v*) A/B. In this case, 5.0 µL of samples or standards were injected (at 10 °C). The QqQ was operated in multiple reaction monitoring (MRM) mode and positive ionization. Two transitions were monitored (Table 4) and a maximum tolerance of ±20% was set for the relative intensities of precursor and product ions [45]. The rest of the parameters for the MS were set as follows: capillary voltage, 3.5 kV, desolvation temperature, 500 °C; source temperature, 150 °C, desolvation gas flow (N_2_), 900 L/h, cone gas flow (N_2_), 50 L/h; collision gas pressure (Ar), 0.5 bar.

In both instruments, water and ACN with 0.1% *v*/*v* formic acid were used as aqueous (A) and organic (B) mobile phases, respectively. The same column (Acquity UPLC^®^ BEH C_18_, 50 mm × 2.1 mm, 1.7 μm, Waters) and pre-column (Acquity UPLC^®^ BEH C_18_, 5 mm × 2.1 mm, 1.7 μm, Waters) were also employed in both instruments. MassLynx software (Waters Chromatography) was used to control the instruments (pumps, sample manager and MS parameters), data acquisition and processing.

### 3.3. NaHDES Synthesis and VA-LLME

The synthesis of the NaHDESs used in the subsequent LLME protocol was made according to our previous experience [8,9]. Suitable amounts (molar ratio 2:1) of Th and OctA were mixed in screw-capped glass tubes and heated (80 °C) and stirred (850 rpm) for 10 min. As a result, homogeneous transparent DES liquids were obtained. For VA-LLME, 125 µL of the NaHDES were rapidly injected into 10 mL of kombucha, previously adjusted to pH 6.00 (with NaOH 0.1 M or 6 M or HCl 0.1 M solutions) and spiked with ISs (at 25 µg/L). After vortexing for 1 min, the mixture was centrifuged (3000 rpm ―2465× *g*―, 10 min, 15 °C) in an Eppendorf 5810R centrifuge (Hamburg, Germany). Ten µL of the floating drop were recovered and diluted 20-fold with ACN for injection.

### 3.4. Samples Slection

A total of 26 different varieties of kombucha from nine different brands (Table S1) were purchased in supermarkets and/or organic shops from Tenerife (Canary Islands, Spain). These samples had the EU organic product label and were bottled in glass containers with plastic caps (19) or in plastic bottles (7). The samples had different fruity (K5, K7, K8, K10-K13, K15-K18, K20-K24 and K26) and herbal (K3, K4, K14) flavors. Among them, two varieties are declared as zero sugar (manufacturer indicates on the label that the sugar is fully consumed during fermentation). It should be noted that one variant (K19) contained an algae (spirulina) among its ingredients.

The pH of the original samples varied between 2.80 and 3.25, as expected for acidic beverages. The nutritional information (g/100 g, according to their labels) indicated that carbohydrates varied between 0.0 and 4.5, fats ranged between 0.0 and 0.5 and proteins oscillated in the range 0.0–0.7.

A plain kombucha (without additional flavors) was selected to validate the methodology. For this purpose, that sample was analyzed to check for PAEs content and no residues were detected. In order to separate SCOBY from the liquid phase, the samples were centrifuged (3400 rpm ―2326× *g*― for 10 min) and filtered (0.45 µm filters PVDF, Millipore, Sigma-Aldrich). Finally, the samples were degassed (magnetic stirring for 10 min) before pH adjustment. It should be noted that the sample portion considered for analysis was selected on the basis of the end-user’s consumption pattern. Kombucha producers separate the SCOBY from the liquor before bottling it [12], thus a reduced sedimented phase is incorporated in the commercial format (e.g. 0.0413 ± 0.011% *w*/*v* for the kombucha used during validation).

## 4. Conclusions

A VA-LLME-UHPLC-MS method [9] based on the NaHDES Th:OctA molar ratio 2:1 has been used for the analysis of a group of 11 PAEs (DMP, DMEP, DEP, DPP, BBP, DBP, DBEP, DNPP, DCHP, DEHP and DNOP) and one adipate (DEHA) in 26 varieties of kombuchas (19 samples bottled in glass and 7 samples bottled in plastic). Among these samples, different varieties with fruity and/or herbal flavors were included. Only DMP and DBP were detected in 11 samples, although frequently at concentrations lower than the LOQ. Almost all the kombuchas bottled in plastic present these PAEs. However, despite being only present in five of the 19 samples in glass bottles, the highest concentration was detected for DMP (16.18 ± 1.14 µg/L) in one of these matrices. Although this aspect was certainly contrary to what was expected (higher concentration of phthalates in plastic-bottled samples), only 26% of the samples bottled in glass presented PAEs vs. 85% of the analyzed samples packaged in plastic. The presence of these compounds in samples bottled in glass could be due to the plastic materials in the manufacturing line or because of the caps in contact with the kombuchas (acidic beverages facilitate the migration of DMP and DBP). These results show the need to assess foods/drinks in which these substances are not expected to be found. It should be mentioned that the estimated daily intake for DMP in kombucha (0.36 µg/kg-bw/d) was higher than the obtained for beverages in China (0.01 µg/kg-bw/d) and Europe (0.00 µg/kg-bw/d). Finally, it should be remarked that the method employed is green (use of small volumes of a natural and sustainable solvent), quick, easy, and provides good extraction efficiency. To our knowledge, this paper reports for the first time the analysis of these plastic migrants in kombucha.

## Figures and Tables

**Table 1:** Matrix effect (ME), Mandel’s test and matrix-matched calibration data (area/area_IS_ vs. concentration) for the selected PAEs and DEHA.

Analyte	ME % (RSD, %)	Mandel’s Test	Calibration Data (*n* = 8)				
		*F_exp_* *	Range of Concentration ^#^ (µg/L)	Regression Equation	R^2^	e	E (10^−2^)
DMP	16 (1)	592.87	1.25–62.50	y = (1.52 ± 0.16)·10^−4^ x^2^ + (4.23 ± 1.02)·10^−3^ x + (2.11 ± 1.13)·10^−2^	0.9996	3.03·10^−4^ x + 4.23·10^−3^	1.05
DMEP	13 (17)	6885.32	18.75–62.50	y = (5.02 ± 1.33)·10^−5^ x^2^ − (2.63 ± 1.08)·10^−3^ x + (4.95 ± 1.97)·10^−2^	0.9999	1.00·10^−4^ x − 2.63·10^−3^	0.16
DEP	39 (7)	90.01	6.25–62.50	y = (1.46 ± 0.60)·10^−4^ x^2^ − (0.29 ± 4.24)·10^−3^ x + (0.43 ± 5.42)·10^−2^	0.9981	2.92·10^−4^ x − 2.85·10^−4^	0.82
DPP	17 (3)	63.94	1.25–62.50	y = (2.71 ± 0.87)·10^−4^ x^2^ + (3.30 ± 5.59)·10^−3^ x + (3.98 ± 6.16)·10^−2^	0.9952	5.42·10^−4^ x + 3.30·10^−3^	1.46
BBP	19 (1)	71.67	12.50–62.50	y = (8.56 ± 6.11)·10^−6^ x^2^ + (2.66 ± 4.75)·10^−4^ x − (0.75 ± 7.41)·10^−3^	0.9991	1.71·10^−5^ x + 2.66·10^−4^	0.08
DBP	17 (5)	43.41	18.75–62.50	y = (0.90 ± 1.04)·10^−4^ x^2^ + (0.20 ± 8.69)·10^−3^ x + (0.20 ± 1.53)·10^−1^	0.9999	1.81·10^−4^ x + 1.96·10^−4^	0.54
DBEP	18 (4)	67.62	2.50–62.50	y = (1.76 ± 0.65)·10^−5^ x^2^ + (1.22 ± 4.36)·10^−4^ x + (2.49 ± 5.30)·10^−3^	0.9957	3.51·10^−5^ x + 1.22·10^−4^	0.10
DNPP	23 (4)	296.54	1.25–62.50	y = (3.38 ± 0.51)·10^−4^ x^2^ + (4.17 ± 3.24)·10^−3^ x + (3.89 ± 3.57)·10^−2^	0.9990	6.77·10^−4^ x + 4.17·10^−3^	1.82
DEHP	15 (3)	123.52	1.25–62.50	y = (9.04 ± 2.09)·10^−5^ x^2^ + (1.41 ± 1.34)·10^−3^ x + (0.98 ± 1.48)·10^−2^	0.9977	1.81·10^−4^ x + 1.41·10^−3^	0.52
DCHP	15 (16)	60.28	1.25–62.50	y = (6.71 ± 2.22)·10^−6^ x^2^ + (0.30 ± 1.42)·10^−4^ x + (2.38 ± 1.57)·10^−3^	0.9938	1.34·10^−5^ x + 3.01·10^−5^	0.03
DEHA	245 (2)	171.98	1.25–62.50	y = (1.79 ± 0.35)·10^−4^ x^2^ + (1.15 ± 0.23)·10^−2^ x + (1.76 ± 0.25)·10^−1^	0.9994	3.58·10^−4^ x + 1.15·10^−2^	1.89
DNOP	66 (9)	26.56	12.75–62.50	y = (2.03 ± 2.35)·10^−4^ x^2^ + (1.30 ± 1.83)·10^−2^ x − (1.81 ± 2.85)·10^−1^	0.9985	4.05·10^−4^ x + 1.30·10^−2^	2.57

* *F_tab_* _(α=0_._05, 1, *n*−3=8)_ = 6.61; ^#^ sample concentration.

**Table 2:** Recovery values (*n* = 5), repeatability (*n* = 5) and LODs and LOQs of the VA-LLME-UHPLC-MS method in kombuchas.

Analyte	Recovery Study (*n* = 5)				Repeatability (*n* = 5)		LOD (µg/L)	LOQ (µg/L)
	Concentration (µg/L)	Recovery, % (RSD, %)	Concentration (µg/L)	Recovery, % (RSD, %)	Concentration (µg/L)	RSD, %		
DMP	2.5	100 (6)	50	110 (3)	50	7	0.21	0.70
DMEP	18.75	117 (2)	50	116 (3)	50	4	5.45	18.15
DEP	2.5	112 (8)	50	118 (3)	50	6	1.84	6.14
DPP	2.5	115 (6)	50	120 (6)	50	4	0.12	0.40
BBP	18.75	113 (9)	50	114 (15)	50	4	3.02	10.08
DBP	18.75	120 (4)	50	113 (12)	50	2	2.93	9.77
DBEP	2.5	119 (11)	50	112 (14)	50	3	0.61	2.04
DNPP	18.75	96 (13)	50	115 (12)	50	10	0.07	0.24
DEHP	2.5	110 (19)	50	115 (9)	50	9	0.09	0.29
DCHP	2.5	67 (16)	50	117 (11)	50	6	0.26	0.86
DEHA	2.5	99 (19)	50	114 (13)	50	5	0.25	0.82
DNOP	18.75	107 (7)	50	107 (16)	50	3	3.31	11.04

**Table 3:** Results of the analysis of kombucha samples using the NaHDES-VA-LLME-UHPLC-MS/MS method.

Sample	Analite Concentration (µg/L) (*n* = 5)	
	DMP	DBP
K2	16.18 ± 1.14	-
K4	<LOQ	-
K9	3.22 ± 2.24	-
K10	3.55 ± 2.17	-
K15	4.11 ± 2.34	-
K20	<LOQ	<LOQ
K21	-	<LOQ
K22	<LOQ	<LOQ
K23	<LOQ	<LOQ
K24	7.68 ± 1.56	<LOQ
K25	<LOQ	<LOQ

**Table 4:** MS and MS/MS parameters of the selected plasticizers and ISs.

Analyte	SIM			MRM						
	Rt (min)	Ion (m/z)	Cone Voltage (V)	Rt (min)	Quantification Transition (m/z)	Cone Voltage (V)	Collision Energy (V)	Qualification Transition (m/z)	Cone Voltage (V)	Collision Energy (V)
DMP	0.79	162.9	16	0.56	195.0 → 76.9	16	32	195.0 → 162.9	16	8
DMEP	0.81	283.1	14	0.55	283.1 → 58.9	14	16	283.1 → 207.0	14	6
DEP	1.30	176.9	16	0.63	223.1 → 148.9	16	18	223.1 → 176.9	16	8
DPP	2.45	148.9	18	0.76	251.1 → 148.9	18	16	251.1 → 191.0	18	6
BBP	3.84	205.0	22	0.93	313.4 →90.9	22	16	313.4 → 205.0	22	8
DBP	4.09	279.1	14	1.03	279.1 → 148.9	14	12	279.1 → 205.0	14	6
DBEP	4.18	367.2	18	1.01	367.2 → 44.9	18	24	367.2 → 54.6	18	16
DNPP	5.67	148.9	22	1.50	307.2 → 148.9	22	14	307.2 → 219.0	22	8
DEHP	5.72	166.9	22	2.91	391.3 → 113.0	22	8	391.3 → 166.9	22	12
DCHP	5.78	331.2	22	1.60	331.2 → 148.9	22	26	331.2 →166.9	22	12
DEHA	8.20	129.0	24	2.93	371.4 → 110.9	24	24	371.4 → 129.0	24	16
DNOP	8.35	148.9	22	3.04	391.4 → 92.9	22	58	391.4 → 148.9	22	22
DBP-d_4_	3.96	153.0	20	1.03	283.2 →153.0	20	12	283.2 → 209.0	20	8
DHP-d_4_	6.84	237.1	22	2.11	339.3 → 153.0	22	12	339.3 → 237.1	22	8

## Data Availability

Data supporting reported results are included in this document.

## Supplementary Materials

The following supporting information can be downloaded. Table S1: Characteristics of the analyzed samples.
