# Supplementary material for: Application of a Liquid-Liquid Microextraction Method Based on a Natural Hydrophobic Deep Eutectic Solvent for the Extraction of Plastic Migrants from Kombuchas
Source: Molecules. 2021 Dec 28;27(1):178. doi: 10.3390/molecules27010178 (PMC8746289; doi:10.3390/molecules27010178)
Supplement: Supplementary file 1 [file molecules-27-00178-s001.zip › molecules-1487315-supplementary.pdf]

Supplementary Material. Application of a liquid-liquid microextraction method based on a natural hydrophobic deep eutectic solvent for the extraction of plastic migrants from kombuchas

Antonio V. Herrera-Herrera [1,2], Ruth Rodríguez-Ramos [1], Álvaro Santana-Mayor [1], Bárbara Socas-Rodríguez [1], and Miguel Ángel Rodríguez-Delgado [1]

[1]*Departamento de Química, Unidad Departamental de Química Analítica, Facultad de Ciencias, Universidad de La Laguna (ULL). Avenida Astrofísico Francisco Sánchez, s/nº. 38206 San Cristóbal de La Laguna (Tenerife), España.*

[2]*Instituto Universitario de Bio-Orgánica Antonio González. Universidad de La Laguna (ULL). Avda. Astrofísico Fco. Sánchez, 2. 38206 San Cristóbal de La Laguna, España.*

Table S1. Characteristics of the analyzed samples

Sample	Brand	Bottling format	Flavor	Ingredients	pH	Nutritional facts		
						Carbohydrate content (g/100 g)	Fat content (g/100 g)	Protein content (g/100 g)
K1	A	Glass with plastic cap	Original	Water, cane sugar, green tea	3.10	1.8	<0.1	0.06
K2	A	Glass with plastic cap	Ginger & turmeric	Water, cane sugar, ginger, turmeric, lemon grass, lemon, green tea, black pepper and stevia infusion	3.20	0.3	<0.1	<0.02
K3	Ai	Glass with plastic cap	Hibiscus	Water, cane sugar, grenade, hibiscus blossom and green tear	2.88	0.2	0.1	0.7
K4	B	Glass with plastic cap	Green tea	Water, cane sugar and green tea	2.88	3.6	0.4	0.2
K5	B	Glass with plastic cap	Red fruits	Water, cane sugar, green tea, strawberry, blueberry and hibiscus blossom	2.85	3.2	0.3	<0.1
K6	B	Glass with plastic cap	Mojito	Water, cane sugar, green tea, lemon, lime and peppermint	2.80	3.0	0.2	<0.3
K7	B	Glass with plastic cap	Ginger & lemon	Water, cane sugar, green tea, ginger and lemon	2.89	1.6	0.5	0.1
K8	B	Glass with plastic cap	Apple, cinnamon &vanilla	Water, cane sugar, green tea, apple, cinnamon and vanilla	2.89	2.8	0.5	<0.08
K9	C	Glass with plastic cap	Original	Water, green tea, black tea, yerba mate leaves, linden blossoms, lemon verbena, acanthus blossoms, lemon balm, nanah mint, nettle, rubella, elderflowers, raspberry leaves, marigold flowers, blackberry leaves, licorice root and cane sugar	3.19	4.3	<0.5	<0.5
K10	C	Glass with plastic cap	Sour cherry & mint	Water, green tea, black tea, yerba mate leaves, linden blossoms, lemon verbena, acanthus blossoms, lemon balm, nanah mint, nettle, rubella, elderflowers, raspberry leaves, marigold flowers, blackberry leaves, licorice root, cane sugar, sour cherry, grape syrup, mint and lemon juice	3.24	4.6	<0.5	<0.5

K11	C	Glass with plastic cap	Lime & ginger	Water, green tea, black tea, yerba mate leaves, linden blossoms, lemon verbena, acanthus blossoms, lemon balm, nanah mint, nettle, rubella, elderflowers, raspberry leaves, marigold flowers, blackberry leaves, licorice root, cane sugar, lime juice, and ginger juice	2.90	4.3	<0.5	<0.5
K12	D	Glass with plastic cap	Ginger & apple	Water, cane sugar, ginger, apple juice and green tea	2.87	1.6	<0.1	<0.3
K13	D	Glass with plastic cap	Hibiscus & pomegranate	Water, cane sugar, pomegranate juice, hibiscus infusion and green tea	2.81	0.2	0.0	0.7
K14	D	Glass with plastic cap	Cucumber & lemon verbena	Water, cane sugar, cucumber, lemon verbena, green tea,	2.85	<0.5	<0.1	<0.3
K15	E	Glass with plastic cap	Açai & red fruits	Water, green tea, cane sugar, acai, hibiscus, apple, rose hip, strawberry and red fruit natural aroma	2.80	3.5	<0.1	<0.1
K16	E	Glass with plastic cap	Mango & strawberry	Water, green tea, cane sugar, red tea, mango and strawberry	2.93	3.7	<0.1	<0.1
K17	F	Glass with plastic cap	Apple & pear	Water, sugar, dextrose, tea, apple juice, citric acid, natural flavoring, ascorbic acid	2.92	4.2	0.0	0.0
K18	G	Glass with plastic cap	Raspberry, strawberry & camu-camu	Water, cane sugar, green tea, strawberry, raspberry, erytrithol, steviol glycosides, camu-camu	2.86	3.7	<0.2	<0.2
K19	G	Glass with plastic cap	Yuzu, spirulina & mint	Water, cane sugar, green tea, yuzu, lemon juice, mint, erytrithol, steviol glycosides, spirulina	2.82	3.6	<0.2	<0.2
K20	H	Plastic	Cranberry	Water, tea, cranberry juice, beet sugar and multifruit juice (aronia, apple, elderberry, lemon, acerola),	3.08	4.5	<0.5g	<0.5
K21	H	Plastic	Lemon & ginger	Water, tea, lemon juice, beet sugar, ginger juice, apple juice and ginger extract	2.98	4.4	<0.5	<0.5
K22	I	Plastic	Passion fruit (zero)	Water, cane sugar*, green tea, stevia leaves, natural passion fruit flavor	3.14	0.0	0.0	0.0
K23	I	Plastic	Raspberry (zero)	Water, cane sugar*, green tea, stevia leaves, natural raspberry flavor and carrot concentrate	3.25	0.0	0.0	0.0
K24	I	Plastic	Raspberry	Water, cane sugar, green tea, stevia leaves, natural raspberry flavor, and carrot, apple and blackcurrant extracts	3.17	4.4	0.0	0.0
K25	I	Plastic	Original	Water, cane sugar, green tea, stevia leaves and tea extract	3.18	4.4	0.0	0.0
K26	I	Plastic	Ginger & lemon	Water, cane sugar, green tea, stevia leaves, natural ginger flavor and natural lemon flavor	3.09	4.4	0.0	0.0

*totally consumed during fermentation
